# Clustering of Gene Expression Data Based on Shape Similarity

**DOI:** 10.1155/2009/195712

**Published:** 2009-03-04

**Authors:** Travis J Hestilow, Yufei Huang

**Affiliations:** 1 Department of Electrical and Computer Engineering, The University of Texas at San Antonio, San Antonio, TX 78249, USA; 2 Greehey Children's Cancer Research Institute, University of Texas Health Science Center at San Antonio, TX 78229, USA

## Abstract

A method for gene clustering from expression profiles using shape information is presented. The conventional clustering approaches such as K-means assume that genes with similar functions have similar expression levels and hence allocate genes with similar expression levels into the same cluster. However, genes with similar function often exhibit similarity in signal shape even though the expression magnitude can be far apart. Therefore, this investigation studies clustering according to signal shape similarity. This shape information is captured in the form of normalized and time-scaled forward first differences, which then are subject to a variational Bayes clustering plus a non-Bayesian (Silhouette) cluster statistic. The statistic shows an improved ability to identify the correct number of clusters and assign the components of cluster. Based on initial results for both generated test data and *Escherichia coli* microarray expression data and initial validation of the *Escherichia coli* results, it is shown that the method has promise in being able to better cluster time-series microarray data according to shape similarity.

## 1. Introduction

Investigating the genetic structure and metabolic functions of organisms is an important yet demanding task. Genetic actions, interactions, how they control and are controlled, are determined, and/or inferred by data from many sources. One of these sources is time-series microarray data, which measure the dynamic expression of genes across an entire organism. Many methods of analyzing this data have been presented and used. One popular method, especially for time-series data, is gene-based profile clustering [1]. This method groups genes with similar expression profiles in order to find genes with similar functions or to relate genes with dissimilar functions across different pathways occurring simultaneously.

There has been much work on clustering time-series data and clustering can be done based on either similarity of expression magnitude or the shape of expression dynamics. Clustering methods include hierarchical and partitional types (such as K-means, fuzzy K-means, and mixture modeling) [2]. Each method has its strengths and weaknesses. Hierarchical techniques do not produce clusters per se; rather, they produce trees or dendrograms. Clusters can be built from these structures by later cutting the output structure at various levels. Hierarchical techniques can be computationally expensive, require relatively smooth data, and/or be unable to "recover" from a poor guess; that is, the method is unable to reverse itself and recalculate from a prior clustering set. They also often require manual intervention in order to properly delineate the clusters. Finally, the clusters themselves must be well defined. Noisy data resulting in ill-defined boundaries between clusters usually results in a poor cluster set.

Partitional clustering techniques strive to group data vectors (in this case, gene expression profiles) into clusters such that the data in a particular cluster are more similar to each other than to data in other clusters. Partitional clustering can be done on the data itself or on spline representations of the data [3, 4]. In either case, square-error techniques such as K-means are often used. K-means is computationally efficient and can always find the global minimum variance. However, it must know the number of clusters in advance; there is no provision for determining an unknown number of clusters other than repeatedly testing the algorithm with different cluster numbers, which for large datasets can be very time consuming. Further, as is the case with hierarchical methods, K-means is best suited for clusters which are compact and well separated; it performs poorly with overlapping clusters. Finally, it is sensitive to noise and has no provision for accounting for such noise through a probabilistic model or the like. A related technique, fuzzy K-means, attempts to mimic the idea of posterior cluster membership probability through a concept of "degree of membership." However, this method is not computationally efficient and requires at least an a priori estimate of the degree of membership for each data point. Also, the number of clusters must be supplied a priori, or a separate algorithm must be used in order to determine the optimum number of clusters. Another similar method is agglomerative clustering [5]. Model-based techniques go beyond fuzzy K-means and actually attempt to model the underlying distributions of the data. The methods maximize the likelihood of the data given the proposed model [4, 6].

More recently, much study has been given toward clustering based on expression profile shape (or trajectory) rather than absolute levels. Kim et al. [7] show that genes with similar function often exhibit similarity in signal shape even though the expression magnitude can be far apart. Therefore, expression shape is a more important indication of similar gene functions than expression magnitude.

The same clustering methods mentioned above can be used based on shape similarity. An excellent example of a tree-based algorithm using shape-similarity as a criterion can be found in [8]. While the results of this investigation proved fruitful, it should be noted that the data used in the study resulted in well-defined clusters. Further, the clustering was done manually once the dendrogram was created. Möller-Levet et al. [9] used fuzzy K-means to cluster time-series microarray data using shape similarity as a criterion. However, the number of clusters was known beforehand; no separate optimization method was used in order to find the proper number of clusters. Balasubramaniyan et al. [10] used a similarity measure over time-shifted profiles to find local (short-time scale) similarities. Phang et al. [11] used a simple  shape decomposition and used a nonparametric Kruskal-Wallis test to group the trajectories. Finally, Tjaden [12] used a K-means related method with error information included intrinsically in the algorithm.

A common difficulty with these approaches is to determine the optimal number of clusters. There have been numerous studies and surveys over the years aimed at finding optimal methods for unsupervised clustering of data; for example, [13–20]. Different methods achieve different results, and no single method appears to be optimal in a global sense. The problem is essentially a model selection problem. It is well known that the Bayesian methods provide the optimal framework for selecting models, though a complete treatment is analytically intractable for most cases. In this paper, a Bayesian approach based on the Variational Bayes Expectation Maximization (VBEM) algorithm is proposed to determine the number of clusters and better performance than MDL and BIC criterion has been demonstrated.

In this study, the goal was to find clusters of genes with similar functions; that is, coregulated genes using time-series microarray data. As a result, we choose to cluster genes based on signal shape information. Particularly, signal shape information is derived from the normalized time-scaled forward first differences of the time-sequence data. This information is then forwarded to a Variational Bayes Expectation Maximization algorithm (VBEM, [21]), which performs the clustering. Unlike K-means, VBEM is a probabilistic method, which was derived based on the Bayesian statistical framework and has shown to provide better performance. Further, when paired with an external clustering statistic such as the Silhouette statistic [22], the VBEM algorithm can also determine the optimal number of clusters.

The rest of the paper is organized as follows. In Section 2 the problem is discussed in more detail, the underlying model is developed, and the algorithm is presented. In Section 3 the results of our evaluation of the algorithm against both simulated and real time-series data are shown. Also presented are comparisons between the algorithm and K-means clustering, both methods using several different criteria for making clustering decisions. Conclusions are summarized in Section 4. Finally, Appendices A, B, and C present a more detailed derivation of the algorithm.

## 2. Method

### 2.1. Problem Statement and Method

Given the microarray datasets of  genes,  for , where  is the number of time points, that is, the columns in the microarray, it is desired to cluster the gene expressions based on signal shape. The clustering is not known a priori; therefore not only must individual genes be assigned to relevant clusters, but the number of clusters themselves must also be determined.

The clustering is based on expression-level shape rather than magnitude. The shape information is captured by the first-order time difference. However, since the gene expression profiles were obscured by the varying levels manifested in the data, the time difference must be obtained on the expression levels with the same scale and dynamic range. Motivated by the observations, the proposed algorithm has three steps. In the first step, the expression data is rescaled. In the second step, the signal shape information is captured by calculating the first-order time difference. In the last step, clustering is performed on the time-difference data using a Variational Bayes Expectation Maximization (VBEM) algorithm. In the following, each step is discussed in detail.

### 2.2. Initial Data Transformation

Each gene sequence was rescaled by subtracting the mean value of each sequence from each individual gene, resulting in sequences with zero mean. This operation was intended to mitigate the widely different magnitudes and slopes in the profile data. By resetting all genes to a zero-mean sequence, the overall shape of each sequence could be better identified without the complication of comparing genes with different magnitudes.

After this, the resulting sequences were then normalized such that the maximum absolute value of the sequence was 1. Gene expression between related genes can result in a large change or a small; if two genes are related, that relationship should be recoverable regardless of the amplitude of change. By renormalizing the data in this manner, the amplitudes of both large-change and small-change genes were placed into the same order of magnitude.

Mathematically, the above operation can be expressed by (1)

where  represents the mean of .

### 2.3. Extraction of Shape Information and Time Scaling

To extract shape information of time-varying gene expression, the derivative of the expression trajectory is considered. Since we are dealing with discrete sequences, differences must be used rather than analytical derivatives. To characterize the shape of each sequence, a simple first-difference scheme was used, this being the magnitude difference of the succeeding point and the point under consideration, divided by the time difference between those points. The data was taken nonuniformly over a period of approximately 100 minutes, with sample times varying from 7 to 50 minutes. As the transformation in (1) already scales the data to a range of , further compressing that scale by nearly 2 orders of magnitude over some time stretches was deemed neither prudent nor necessary. Therefore, the time difference was scaled in hours to prevent this unneeded range compression. The resulting sequences were used as data for clustering.

Mathematically, this operation can be written as (2)

where  is the length- vector of time points associated with gene ,  is the vector of transformed time-series data (from (1)) associated with gene , and  is the resulting vector of first differences associated with gene .

**Figure 1:**
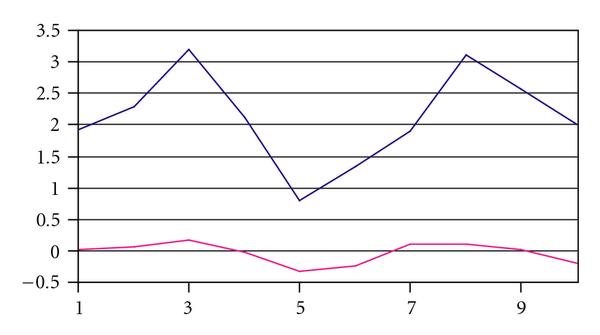
**Dissimilar expression levels with similar shape**.

**Figure 2:**
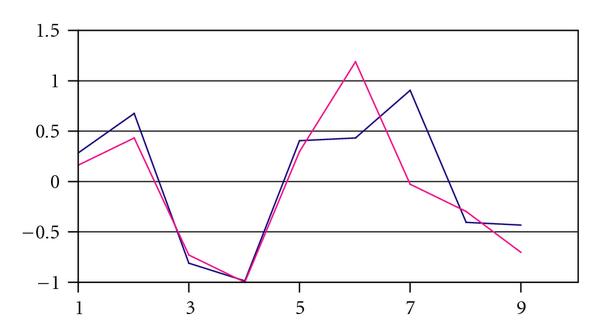
**Normalized differences: the same two sequences after transformations**.

Figure 1 shows an example pair of sequences using contrived data. These two sequences are visually related in shape, but their mean values are greatly different. A K-means clustering would place these two sequences in different clusters. By transforming the data, the similarity of the two sequences is enhanced, and the clustering algorithm can then place them in the same cluster. Figure 2 shows the original two sequences after data transformation.

### 2.4. Clustering

Once the sequence of first differences was calculated for each gene, clustering was performed on , the first-order difference. To this end, a VBEM algorithm was developed. Before presenting that development, a general discussion of VBEM is in order.

An important problem in Bayesian inference is determining the best model for a set of data from many competing models. The problem itself can be stated fairly compactly. Given a set of data , the marginal likelihood of that data given a particular model *m* can be expressed as(3)

where  and  are, respectively, the latent variables and the model parameters. The integration is taken over both variables and parameters in order to prevent overfitting, as a model with many parameters would naturally be able to fit a wider variety of datasets than a model with few parameters.

Unfortunately, this integral is not easily solved. The VBEM method approximates this by introducing a free distribution, , and taking the logarithm of the above integral. If  has support everywhere that  does, we can construct a lower bound to the integral using Jensen's inequality:(4)

Maximizing this lower bound with respect to the free distribution  results in , the joint posterior. Since the normalizing constant is not known, this posterior cannot be calculated exactly. Therefore another simplification is made. The free distribution  is assumed to be factorable, that is, . The inequality then becomes(5)

Maximizing this functional  is equivalent to minimizing the KL distance between  and . The distributions  and  are coupled and must be iterated until they converge.

With the above discussion in mind, we now develop the model that our VBEM algorithm is based on. Given  clusters in total, we can let  denote the cluster number of gene . Then, we assume that, given , the expression level for gene *g* follows a Gaussian distribution, that is, (6)

where  is the mean and  is the variance of the *k*th Gaussian cluster. Since both  and  are unknown parameters, a Normal-Inverse-Gamma prior distribution is assigned as(7)

where , , and  are the known parameters of the prior distribution. Furthermore, a multinomial prior is assigned for the cluster number  as (8)

where  is the prior probability that gene  belongs to th cluster  and .  further assumes a priori the Dirichlet distribution(9)

where  are the known parameters of the distribution. Given the transformed expressions of  genes, , the stated two tasks are equivalent to estimating , the total number of clusters, and  for all  genes.

A Bayesian framework is adopted for estimating both  and , which are calculated by the maximum a posteriori criterion as(10)

where  is the marginal likelihood given the model  has  clusters, and  is the *a posteriori* probability of  when the total number of clusters is .

Unfortunately, there are now multiple unknown nuisance parameters at this point: , , , , , and  all still need to be found. To do so requires a marginalization procedure over all the unknowns, which is intractable for unknown cluster id . Therefore, a VBEM scheme is adopted for estimating the necessary distributions.

### 2.5. VBEM Algorithm

Given the development above,  can be expressed as (11)

where  is the vector of unknown parameters , , , , , and . Notice the summation in (11) is NP hard, whose complexity increases exponentially with the number of genes. We therefore resort to approximate this integration by variational EM. First, a lower bound is constructed for the expression in (11). The ultimate aim is to maximize this lower bound. The expression for the lower bound can be written(12)

where as above the inequality derives by use of Jensen's inequality. The free distributions  and  are introduced as approximations to the unknown distributions  and . The  distributions are chosen so as to maximize the lower bound. Using variational derivatives and an iterative coordinate ascent procedure, we find

Vbe Step:(13)

Vbm Step:(14)

where  and  are iterations and  are normalizing constants to be determined. Because of the integration in (13),  must be chosen carefully in order to have an analytic expression. By choosing  as a member of the exponential family, this condition is satisfied. Note  is an approximation to the posterior distribution  and therefore can be used to obtain the estimate of .

### 2.6. Summary of VBEM Algorithm

The VBEM algorithm is summarized as follows:

(1) Initialization

(i) Initialize , , **a**, **b**, **k**, and **L**.

Iterate until lower bound converges enumerate

(2) VBE Step:

(i) for ,

(ii) calculate  using (A.1) in Appendix A,

(iii) end .

(3) VBM Step:

(i) for ,

(ii) calculate  using (B.1) in Appendix B,

(iii) End *k*.

(4) Lower bound:

(i) calculate  using (C.1) in Appendix C.

 End iteration.

### 2.7. Choice of the Optimum Number of Clusters

The Bayesian formulation of (11) suggests using the number of clusters that maximize the marginal likelihood, or in the context of VBEM, the lower bound . Instead of solely basing the determination of the number of clusters using , 4 different criteria are investigated in this work: (a) lower bound  used within the VBEM algorithm (labelled KL), (b) the Bayes Information Criterion [23], (c) the Silhouette statistic performed on clusters built from transformed data, and (d) the Silhouette statistic performed on clusters built from raw data. The VBEM lower bound  is discussed above; the BIC and Silhouette criteria are discussed below.

### 2.8. Bayes Information Criterion (BIC)

The Bayes Information Criterion (BIC, [23]) is an asymptotic approximation to the *Bayes Factor*, which itself is an average likelihood ratio similar to the maximum likelihood ratio. As the Bayes Factor is often a difficult calculation, the BIC offers a less-intensive approximation. Subject to the assumptions of large data size and exponential-family prior distributions, maximizing the BIC is equivalent to maximizing the integrated likelihood function. The BIC can be written as(15)

where  is the likelihood function of data  given parameters ,  is the size (dimensionality) of parameter set , and  is the sample size. The term  is a penalty term discouraging more complex models.

### 2.9. Silhouette Statistic

The Silhouette statistic (Sil, [22]) uses the squared difference between a data vector and all other data vectors in all clusters. For any particular data vector  belonging to cluster , let  be the average squared difference between data vector  and all other vectors in cluster . Let  be the minimum average squared distance between data vector  and all other vectors of cluster , . Then the Silhouette statistic for data vector  is(16)

It is quickly seen that the range of this statistic is . A value close to 1 means the data vector is very probably assigned to the correct cluster, while a value close to  means the data vector is very probably assigned to the wrong cluster. A value near 0 is a neutral evaluation.

## 3. Results

We illustrate the method using simulated expression data and with microarray data available online.

### 3.1. Simulation Study

In order to test the ability of VBEM to properly cluster data of similar shape but dissimilar mean level, and scale, several datasets were constructed. These datasets were intended to appear as would a set of time-series microarray data. Each consisted of 5 data points in a vector, corresponding to what might be seen from a microarray from a single gene over 5-time samples. Identical assumptions were used to produce these datasets; namely, that the inherent clusters within the data were based upon a mean vector of values for a particular cluster, that each cluster may have subclusters exhibiting a mean shift and/or a scale change from the mean vector, and that the data within a cluster randomly varied about that mean vector (plus any mean shift and scale change). All sets of sample data shared the characteristics shown in Table 1. For example, a test "gene" of cluster "dms" would be a random length-5 vector, drawn from a Gaussian distribution with a mean of  and a particular standard deviation (defined below). This random vector would then be scaled by 0.25 and shifted in value by .

**Table 1:** Basis vectors for clusters in sample datasets.

Cluster	Subcluster	Mean vector	Mean shift	Scale factor
a	a		0	1

b	b		0	1
	bm			1

c	c		0	1
	cs		0	0.25

d	d		0	1
	dms			0.25

e	e		0	1
	em			1
	es		0	0.25
	ems			0.25

The datasets constructed from these basis vectors differed in number of data vectors per subcluster (and thus the total number of data vectors), and the standard deviation used to vary the individual vector values about their corresponding basis vectors. Generally speaking, the standard deviation vectors were constructed to be approximately 25% of the mean vector for the "low-noise" sets, and approximately 50% of the mean vector for the "high-noise" sets.

### 3.2. "Low-Noise" Test Datasets

Two datasets were constructed using standard deviation vectors approximately 25% of the relevant mean vector. Table 2 shows the standard deviation vectors used. Each subcluster in Table 1 was replicated several times, randomly varying about the mean vector in a Gaussian distribution with a standard deviation as shown in Table 2. Test set 1 had 5 replicates per subcluster (e.g., a1–a5, cs1–cs5), resulting in a total set  data vectors. Test set 2 had 99 replicates per subcluster, resulting in a total set  data vectors.

**Table 2:** Standard deviation vectors for clusters in "low-noise" sample datasets.

Cluster	Standard deviation vector
a	
b	
c	
d	
e	

### 3.3. "High-Noise" Test Datasets

Because of the need to test the robustness of the clustering and prediction algorithms in the presence of higher amounts of noise, six datasets were constructed using standard deviation vectors approximately 50% of the relevant mean vector. Table 3 shows the standard deviation vectors used. As with the "low-noise" sets, each subcluster in Table 1 was replicated several times, randomly varying about the mean vector in a Gaussian distribution, this time with a standard deviation as shown in Table 3. Table 4 shows the number of replicates produced for each dataset. For the test data, an added transformation step was accomplished that would normally not be performed on actual data. Since the test data was produced in already clustered form, the vectors (rows) were randomly shuffled to break up this clustering.

**Table 3:** Standard deviation vectors for clusters in "high-noise" sample datasets.

Cluster	Standard deviation vector
a	
b	
c	
d	
e	

**Table 4:** Subcluster replicates and total vector sizes for "high-noise" datasets.

Test set	Total replicates	Total *N*
3	5	55
4	9	99
5	30	330
6	50	550
7	70	770
8	99	1089

### 3.4. Test Types and Evaluation Measures

To evaluate the ability of VBEM to properly cluster the datasets, two test sequences were conducted. First, the data was clustered using VBEM in a "controlled" fashion; that is, the number of clusters was assumed to be known and passed to the algorithm. Second, the algorithm was tested in an "uncontrolled" fashion; that is, the number of clusters was unknown, and the algorithm had to predict the number of clusters given the data. During the uncontrolled tests, a K-means algorithm was also run against the data as a comparison.

The VBEM algorithm as currently implemented requires an initial (random) probability matrix for the distribution of genes to clusters, given a value for . Therefore, for each dataset, 55 trials were conducted, each trial having a different initial matrix.

Also, each trial begins with an initial clustering of genes. As currently implemented, this initialization is performed using a K-means algorithm. The algorithm attempts to cluster the data such that the sum of squared differences between data within a cluster is minimized. Depending on the initial starting position, this clustering may change. In MATLAB, the built-in K-means algorithm has several options available to include how many different trials (from different starting points) are conducted to produce a "minimum" sum-squared distance, how many iterations are allowed per trial to reach a stable clustering, and how clusters that become "empty" during the clustering process are handled. For these tests, the K-means algorithm conducted 100 trials of its own per initial probability matrix (and output the clustering with the smallest sum-squared distance), had a limit of 100 iterations, and created a "singleton" cluster when a cluster became empty.

As mentioned above, the choice of optimum *K* was conducted using four different calculations. The first used the estimate for the VBEM lower bound, the second used the BIC equation. In both cases, the optimum  for a particular trial was that which showed a decrease in value when  was increased. This does not mean the values used to determine the optimum  were the absolute maxima for the parameter within that trial; in fact, they usually were not. The overall optimum  for a particular choice of parameter was the maximum value over the number of trials. The third and fourth criteria made use of the Silhouette statistic, one using the clusters of transformed data and one using the corresponding clusters of raw data. We used the built-in Silhouette function contained within MATLAB for our calculations. To find the optimum , the mean Silhouette value for all data vectors in a clustering was calculated for each value of . The value of  for which the mean value was maximized was chosen as the optimum .

To evaluate the actual clustering, a misclassification rate was calculated for each trial cluster. Since the "ground-truth" clustering was known a priori, this rate can be calculated as a sum of probabilities derived from the original data and the clustering results:(17)

where  is the probability that computed cluster  belongs to a priori cluster  given that  is in fact the correct cluster, and  is the probability of a priori cluster  occurring.  refers to the misclassification rate using statistic  (KL, BIC, both Silhouette) for trial . This rate is in the range  and is equal to 1 only when the number of clusters is properly predicted and those calculated clusters match the a priori clusters. Thus, both under- and overprediction of clusters were penalized.

**Figure 3:**
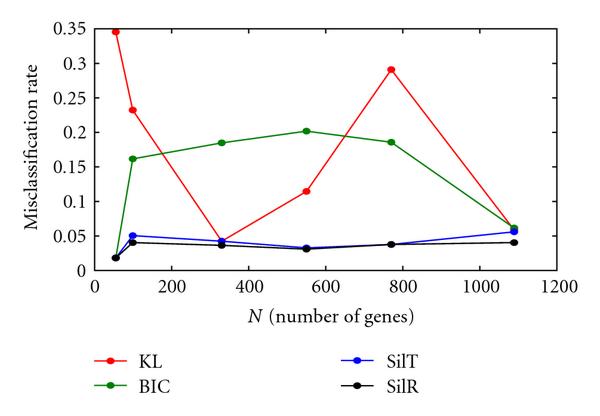
**Misclassification rate versus *N*, high-noise data, *K* fixed**.

For the "controlled" test sequences, the combinations of VBEM + KL (V/KL), VBEM + BIC (V/BIC), VBEM + Silhouette (transformed data) (V/SilT), and VBEM + Silhouette (raw data) (V/SilR) all properly chose the optimum clustering for the two "low-noise" datasets, in all cases with no misclassification. For the six "high-noise" sets, V/KL and V/BIC were completely unable to choose the optimum clustering (lowest misclassification rate). In the case of V/SilT, the algorithm-chosen optimum was rarely the true optimum (2 out of 6 datasets). However, the chosen optimum was always very nearly optimal. Finally, V/SilR chose the optimum clustering 5 out of 6 datasets. The algorithm-chosen optimal clustering for both V/SilT and V/SilR showed a misclassification rate of 6 percent or less, while the misclassification rates for V/KL and V/BIC were often in the range of 15–35 percent. Figure 3 summarizes this data.

For the "uncontrolled" tests, the above 4 algorithms were tested with the number of clusters unknown. Further, K-means clustering with Silhouette statistic (KM/SilT and KM/SilR) was also conducted for comparison. The results for the 6 "high-noise" datasets are summarized below.

**Figure 4:**
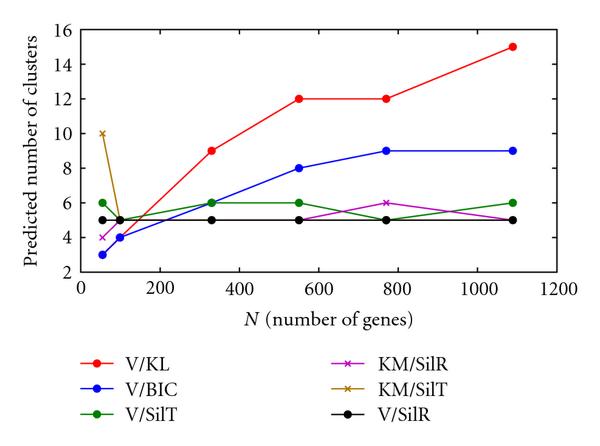
***K*(pred) versus *N*, high-noise data**.

Figure 4 shows a summary plot of the predicted number of clusters *K* versus dataset size *N* for all combinations. Note that V/SilR correctly identified  for all datasets. Also note that KM/SilT, KM/SilR, and V/SilT predicted  or  for all datasets except for test set 3 (). However, even though V/SilR correctly identified  for this dataset, it had equivalent optimum values for , and 15. Given the poor performance of all combinations for this dataset, this suggests that for high-noise data such as this,  is insufficient to give good results.

V/KL and V/BIC both performed poorly with all datasets, in most cases overpredicting the number of clusters. As can be seen in Figure 4, this overprediction tended to increase with dataset size *N*. V/BIC resulted in a lower over-prediction than V/KL.

**Figure 5:**
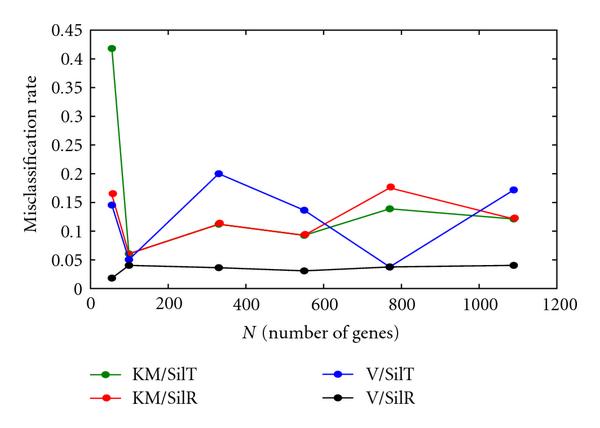
**Misclassification rate versus *N*, high-noise data, *K* unknown**.

Figure 5 shows a summary plot of misclassification rate versus dataset size *N* for the VBEM versus K-means comparison using Silhouette statistics only (both raw and difference). This plot shows the greater performance of V/SilR even more dramatically. While the misclassification rates for the KM/SilT, KM/SilR, and V/SilT were generally on the order of 10–20%, V/SilR was very stable, generally between 3-4%.

### 3.5. Test Results Conclusion

The VBEM algorithm can correctly cluster shape-based data even in the presence of fairly high amounts of noise, when paired with the Silhouette statistic performed on the raw data clusters (V/SilR). Further, V/SilR is robust in correctly predicting the number of clusters in noise. The misclassification rate is superior to K-means using Silhouette statistics, as well as VBEM using all other statistics. Because of this, it was expected that V/SilR would be the algorithm of choice for the experimental microarray data. However, to maintain comparison, all four VBEM/statistic algorithms were tested.

### 3.6. Experimental E. Coli Expression Data

The proposed approach for gene clustering on shape similarity was tested using time-series data from the University of Oklahoma E. coli Gene Expression Database resident at their Bioinformatics Core Facility (OUBCF) [24]. The exploration concentrated on the wild-type MG1655 strain during exponential growth on glucose. The data available consisted of 5 time-series log-ratio samples of 4389 genes.

The initial tests were run against genes identified as being from metabolic categories. Specifically, genes identified in the E. coli K-12 Entrez Genome database at the National Center for Biotechnology Information, US National Library of Medicine, National Institutes of Health (http://www.ncbi.nlm.nih.gov/) [25] (NIH) as being in categories C, G, E, F, H, I, and/or Q were chosen.

Because of the short-sequence lengths, any gene with even a single invalid data point was removed from the set. With only 5-time samples to work with in each gene sequence, even a single missing point would have significant ramifications in the final output. The final set of genes used for testing numbered 1309.

In implementing the VBEM algorithm, initial values for the algorithm were . The algorithm was set to iterate until the change in lower bound decreased below  or became negative (which required the prior iteration to be taken as the end value) or 200 iterations, whichever came first. The optimal number of clusters was arrived at by multiple runs of the algorithm at values of *K*, the predefined number of clusters, varying from 3 to 15.  was chosen in the same manner as in the test data sequences. 

**Figure 6:**
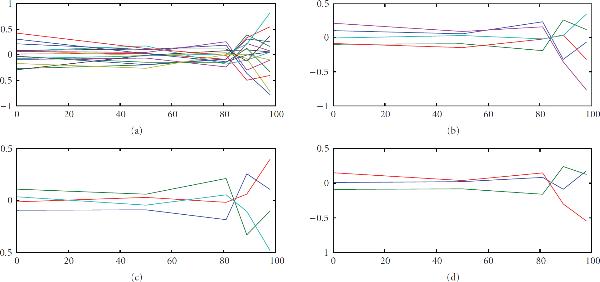
**Mean data shapes**. (a) V/KL, (b) V/BIC, (c) V/SilT, (d) V/SilR.

Figure 6 shows a summary of the final result of the algorithm. Each subfigure shows the mean shapes clustered by the particular algorithm/statistic. As can be seen from the figure, V/KL resulted in an overclassification of structure in the data. The other three algorithms gave more consistent results. As a result of this, the V/KL clusters were removed from further analysis.

### 3.7. Validation of E. Coli Expression Data Results

We validated the results of our tests using Gene Ontology (GO) enrichment analysis. To this end, the genes used in the analysis were tagged with their respective GO categories and analyzed within each cluster for overrepresentation of certain categories versus the "background" level of the population (in this case, the entire set of metabolic genes used). Again, the Entrez Genome database at NIH was used for the GO annotation information. As most of the entries enriched were from the Biological Process portion of the ontology, the analysis was restricted to those terms.

To perform the analysis, the software package Cytoscape (http://www.cytoscape.org/) [26] was used. Cytoscape offers access to a wide variety of plug-in analysis packages, including a GO enrichment analysis tool, BiNGO, which stands for Biological Network Gene Ontology (http://www.psb.ugent.be/cbd/papers/BiNGO/) [27].

To evaluate the clusters, we modified an approach used by Yuan and Li [28] to score the clusters based on the information content and the likelihood of enrichment (). Unlike [28], however, a distance metric was not included in the calculations. Because of the large cluster sizes involved, such distance calculations would have exacted a high calculation overhead. Rather, the simpler approach of forming subclusters of adjacent enriched terms was chosen; that is, if two GO terms had a relationship to each other and were both enriched, they were placed in the same subcluster and their scores multiplied by the number of terms in the subcluster. Also, a large portion of the score of any term shared across more than one cluster was subtracted. This method rewarded large subclusters, while penalizing numerous small subclusters and overlapping terms.

The scoring equation for a cluster , consisting of  subclusters each of size  is given as(18)

where  is the probability of GO term  being selected,  is the negative of the information content of the GO term, and  is the -value  of the GO term . Large subclusters are rewarded by larger values of . Subtracting 1 from  compensates for the "baseline" score value; that is, the score a cluster would achieve if no terms were connected. The final term in the equation is the devaluation of any GO term shared by  clusters.

Given that algorithm was expected to group related functions together, the expectation for GO analysis was the creation of large, highly-connected subclusters within each main gene cluster. Ideally, one such subcluster would subsume the entire cluster; however, a small number of large subclusters within each cluster would validate the algorithm. The scoring equation (18) greatly rewards large, highly-connected subclusters; in fact, given a cluster, the score is maximized by having all GO terms within that cluster be connected within a single  subcluster.

**Figure 7:**
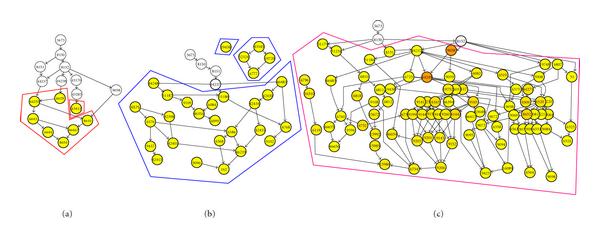
**GO clusters resulting from V/SilR**.

**Figure 8:**
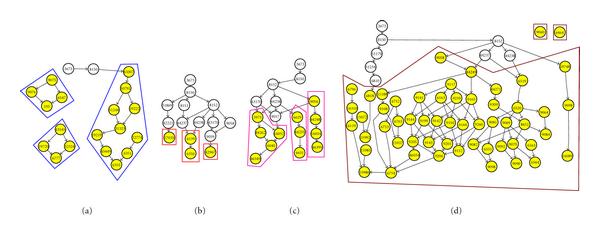
**GO clusters resulting from V/SilT**.

**Figure 9:**
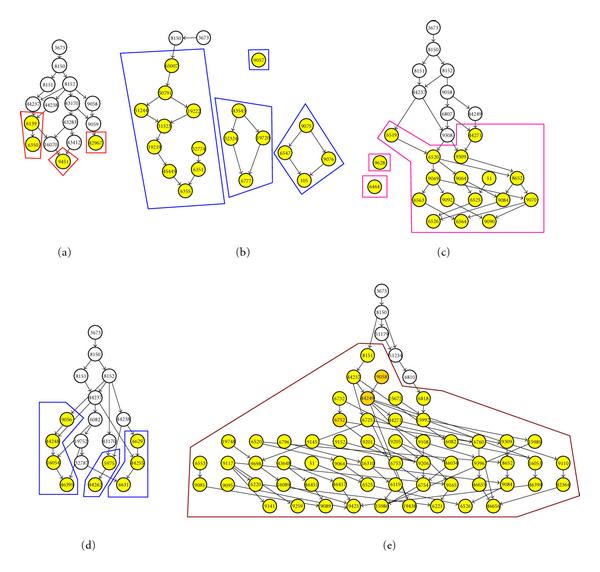
**GO clusters resulting from V/BIC**.

Figures 7, 8, and 9 show the results of the clustering using the three algorithms. Subclusters have been outlined for ease of identification. In some instances, nonenriched GO terms (colored white) have been removed for clarity. Visually, V/SilR is the better choice of the three. It has fewer overall clusters, and each cluster has generally fewer subclusters than V/SilT or V/BIC.

The clusters were scored using (18). Table 5 shows a summary of this analysis. As can be seen, V/SilR (3 clusters) far outscored both V/SilT (4 clusters) and V/BIC (5 clusters), both in aggregate and average cluster scores. Therefore, the conclusion is that V/SilR provides the better clustering performance.

**Table 5:** Summary scores from *E. coli* data analysis

Cluster/algorithm	1	2	3	4	5	Total score	Average score
VSil/R	153.14	2004.55	22129.80			24287.48	8095.83
V/SilT	405.73	3.10	82.95	7343.89		7835.67	1958.92
V/BIC	4.42	422.42	513.70	44.64	11196.16	12181.33	2436.27

## 4. Conclusion

Four combinations of VBEM algorithm and cluster statistics were tested. One of these, VBEM combined with the Silhouette statistic performed on the raw data clusters, clearly outperformed the other three in both simulated and real data tests. This method definitely shows promise in clustering time-series microarray data according to profile shape.

## Appendices

### A. Calculation of VBE Step

Let us assume we are on iteration  and have both  and  available from iteration . Then,(A.1)

where(A.2)

where : number of time samples; : number of genes (index ); , and all other parameters are calculated from the VBM step.

### B. Calculation of VBM Step

Now we assume we have  from the prior VBE step. Then,(B.1)

where ; ; ; ; ; ; : Normal-Inverse-Gamma distribution; : Dirichlet distribution.

### C. Calculation of Lower Bound 

Once  and  have been calculated, we calculate the lower bound using the following:(C.1)(C.2)(C.3)(C.4)

where  and .
